# HDAC2 depletion promotes osteosarcoma’s stemness both in vitro and in vivo: a study on a putative new target for CSCs directed therapy

**DOI:** 10.1186/s13046-018-0978-x

**Published:** 2018-12-03

**Authors:** Marcella La Noce, Francesca Paino, Luigi Mele, Gianpaolo Papaccio, Tarik Regad, Angela Lombardi, Federica Papaccio, Vincenzo Desiderio, Virginia Tirino

**Affiliations:** 1 Dipartimento di Medicina Sperimentale, Sezione di Biotecnologie, Istologia Medica e Biologia Molecolare, Università degli Studi della Campania “L. Vanvitelli”, Napoli, via L. Armanni, 5, 80138 Naples, Italy; 2 Dipartimento di Scienze Biomediche, Chirurgiche e Odontoiatriche, Università degli Studi di Milano, Via Commenda,10, 20122 Milan, Milano Italy; 3 The John van Geest Cancer Research Centre, School of Science and Technology, Nottingham Trent University, Nottingham, UK; 4 Dipartimento di Medicina di Precisione, Università degli Studi della Campania “L. Vanvitelli”, Napoli, Via L. De Crecchio, 7, 80138 Naples, Italy; 5 Dipartimento Medico-Chirurgico di Internistica Clinica e Sperimentale “F. Magrassi”, Università degli Studi della Campania “L. Vanvitelli”, via S. Pansini-Cappella Cangiani, 80131 Naples, Italy

**Keywords:** Cancer stem cells, Osteosarcomas, Methylation, HDAC2, DNMT3a

## Abstract

**Background:**

Cancer stem cells (CSCs) play a key role in cancer initiation, progression and chemoresistance. Epigenetic alterations have been identified as prominent factors that contribute to the CSCs phenotype. Here, we investigated the effects of the HDAC inhibitor valproic acid (VPA) and the demethylating agent, 5’azacytidine (DAC) on the stem phenotype of MG63 and Saos2 osteosarcoma cell lines.

**Methods:**

Saos2 and MG63 cells were treated with DAC and VPA, alone and in combination. Untreated and treated cells were examined for stemness phenotype by cytometry and real-time PCR. Sarcospheres and colonies formation were also evaluated. Moreover, histone modification and methylation were tested by flow cytomery and western blotting. HDAC2 depleted cells were examined for stemness phenotype and their ability to generate tumors in NOD/SCID IL2R-gamma-0 (NSG) mice. HDAC2 expression on human osteosarcoma tissues was evaluated.

**Results:**

We found that DAC and VPA induce an increased expression of stem markers including CD133, OCT4, SOX2 and NANOG, and an increased ability in sarcospheres and colonies formation efficiency. Interestingly, we showed that DAC and VPA treatment decreased repressive histone markers, while increased the active ones. These histone modifications were also associated with an increase of acetylation of histones H3, a decrease of DNA global methylation, HDAC2 and DNMT3a. Furthermore, HDAC2 silenced-MG63 and Saos2 cells acquired a stem phenotype, and promoted in vivo tumorigenesis. In human osteosarcoma tissues, HDAC2 was strongly expressed in nucleus.

**Conclusions:**

Collectively, our results suggest that VPA and DAC induce an expansion of osteosarcoma CSCs, and we report for the first time that HDAC2 is a key factor regulating both CSCs phenotype and in vivo cancer growth. In conclusion, we have identified HDAC2 as a potential therapeutic target in human osteosarcoma treatment.

**Electronic supplementary material:**

The online version of this article (10.1186/s13046-018-0978-x) contains supplementary material, which is available to authorized users.

## Background

Osteosarcoma is one of the most common pediatric tumor in the world after leukemia. The prognosis is poor with 65 and 20% 5-year survival rates for patients with primary or metastatic tumors, respectively. At diagnosis, distant metastases are found in 20% of patients and the incidence of recurrence and metastasis following primary treatment is very high [1, 2]. Although great advances were made in the treatment of this disease, benefits of current therapeutic strategies remain limited, suggesting that new approaches are needed [3]. Osteosarcoma may be considered a disease of osteoblastic differentiation, since regulatory genes, involved in controlling the differentiation of osteoblasts, may be silenced or de-regulated [4]. This could be due to genetic and epigenetic disorders of mesenchymal stem cells that differentiate into osteoblasts [5]. Emerging evidences demonstrated that osteosarcoma contains a subpopulation of cancer stem cells (CSCs) [6–11]; we previously identified a stem cell-like subpopulation expressing CD133 marker, that was able to grow as sarcospheres and form tumors in immunocompromised mice [10, 11].

Recent studies have indicated that specific epigenetic alterations such as DNA methylation and histone modifications could have a key role in generating the CSC phenotype [12–16]. Epigenetic alterations could result in the genesis of aberrant progenitor cells that undergo a series of oncogenic mutations [17]. Therefore, changes in DNA methylation and histones are an important feature of tumor initiation and progression. For instance, these epigenetic modifications such as histone acetylation and DNA methylation can regulate CD133 transcription [18, 19]. Baba et al. showed that ovarian CD133^+^ CSCs could be epigenetically regulated leading to CD133 expression and that it correlates with histones acetylation and DNA methylation [19]. Osteosarcoma is characterized by a significant genomic instability that could lead to a complete rearrangement of a single chromosome and generation of new fusion products [20, 21]. This could be due to interplays with tumor microenvironment, host’s immune system and vascular network that could encourage epigenetic alterations [22–24]. Therefore, it is important to understand the involvement of epigenetics in osteosarcoma initiation, since it could be therapeutically reversible. Here, we examined the epigenetic effects of the HDAC inhibitor valproic acid (VPA) and the demethylating agent, 5-aza-2′-deoxycytidine (DAC) on osteosarcoma cell lines. We found that treatments with VPA and DAC promote the emergence of a CSC population that is characterized by an epigenetic pattern, where DNA hypomethylation, increase of H3 histone acetylation, H3K4me2 and H3K4me3 and decrease of H3K9me3, H3K27me3 and DNMT3a are observed. Finally, knockdown experiments have identified HDAC2 as an essential player in controlling osteosarcoma stemness and tumorigenesis. Collectively, these results support a model by which the modulation of HDAC2 and DNMT3a plays a key role in generating and maintaining the cancer stem phenotype in osteosarcoma.

## Methods

### Cell culture and treatments

Saos2 and MG63 osteosarcoma cell lines were purchased from ATCC CELL BANK; cells were placed in DMEM culture medium, supplemented with 10% FBS, 100 mM 2P-ascorbic acid, 2 mM L-glutamine, 100 U/ml penicillin, 100 mg/ml streptomycin (all purchased from Invitrogen, San Giuliano Milanese, Milan, Italy) and placed in 25 ml flasks with filtered valves. Treatments with 5-aza-2′-deoxycytidine (DAC) (Sigma Aldrich, Milan, Italy) and valproic acid (VPA) (Sigma Aldrich), both diluted in H_2_O, were administered at 3 μM and 0,5 mM, respectively, and in combination replenishing them every 24 h for 48 h.

### MTT analyses

MTT assay (3-(4, 5-dimethylthiazol-2-yl)-2, 5-diphenyltetrazolium bromide) was used to measure the inhibition of cell proliferation and to calculate the IC50. MTT was added in cells exposed to either DAC or VPA at the concentrations of 1,3,5, 10 and 100 μM at 48, 72 and 96 h for DAC and 0,5 and 1 mM at 48, 72 and 96 h for VPA. After identifying non-cytotoxic concentrations, we performed an MTT assay to evaluate the combined effect of both drugs at 48,72 and 96 h.

Four hours later, the formazan precipitate was dissolved in 100 μL dimethyl sulfoxide, and then the absorbance was measured in an ELISA reader (Thermo Molecular Devices Co., Union City, USA) at 550 nm.

### Growth analysis

Treated and untreated cells were plated at a density of 10 × 10^4^ cells/well in 6-well plates. Cells were harvested and re-suspended in PBS at 48, 72 and 96 h. An aliquot of cell suspension was diluted with 0.4% trypan blue (Sigma Aldrich), pipetted onto a haemocytometer and counted under a microscope at 200x magnification. Live cells excluded the dye, whereas dead cells admitted the dye and consequently stained intensely with trypan blue. The number of viable cells for each experimental condition was counted and represented on a linear graph.

### Sarcospheres assay

Treated and untreated MG63 and Saos2 cells were plated at a density of 6 × 10^4^ cells/well in 6-well ultra-low-attachment plates (Corning, Corning, NY, USA) in DMEM/F12 cell medium supplemented with progesterone (10 nM), putrescine (50 nM), sodium selenite (15 nM), transferrin (13 ng/ml), insulin (10 ng/ml; Sigma, St. Louis, MO, USA), human EGF (10 ng/ml), and human bFGF (10 ng/ml; Sigma Aldrich). Fresh aliquots of EGF and bFGF were added every day. After 48–72 h from culture, sarcospheres were visible with an inverted phase-contrast microscope (TS 100; Nikon, Milan, Italy). The sarcosphere-forming efficiency (SSFE) was calculated as the number of sphere-like structures formed in 2 days divided by the original number of cells seeded and expressed as a percentage (mean ± SD).

### Flow cytometry

Treated and untreated cells were detached by trypsin, counted and washed in 0.1% BSA in PBS. At least 500,000 cells were incubated with fluorescent-labeled monoclonal antibodies or respective isotype controls (1/10 diluted 4 °C for 30 min in the dark). After washing steps, the labeled cells were analyzed by flow cytometry using a BD FACS Aria III (Becton & Dickinson, Mountain View, CA, USA). The antibodies used were mouse anti-human CD133/2 PE (Miltenyi Biotec S.r.l. Calderara di Reno, Bologna, Italy), mouse anti-human CD29 PerCP-Cy5.5 (BD Pharmingen, Buccinasco, Milan, Italy), mouse anti-human CD44 FITC (BD Pharmingen, Buccinasco, Milan, Italy), and mouse anti-human CD324 PE (BD Pharmingen).

For intracellular and nuclear staining of vimentin, osteocalcin, OCT4 PE, Sox2 FITC and Nanog PerCP-Cy5.5, cells were processed using the Caltag Fix & Perm Kit (Invitrogen, Milan, Italy) following the manufacturer’s guidelines. Isotypes were used as controls. All data were analyzed using a Diva software 6.*6.*

### Histone modification and global DNA methylation evaluation

Treated and untreated cells were detached by trypsin, counted and washed in 0.1% BSA in PBS. At least 500,000 cells were stained using Caltag Fix & Perm Kit (Invitrogen, Milan, Italy) following the manufacturer’s guidelines with some modifications. Briefly, the cells were fixed with medium A for 15 min at room temperature, washed with PBS and permeabilized with 0,5% triton X 100 for one hour at room temperature. After washing steps, the cells were incubated with medium B plus antibodies or respective isotype controls for 1 h at room temperature. The primary antibodies used were: H3trimethyl k9, H3acetyl k9, H3trimethyl k27, H3trimethyl k4, and H3dimethyl k4, and HDAC2 antibodies, all purchased by Abcam. Total methylated DNA was detected by immunolabeling with anti-5-methylcytidine antibody (Abcam). This antibody recognizes 5-methylcytidine, a modified base found in the DNA of plants and vertebrates. It is specific to the methylated base and has minimal reactivity to non-methylated cytidine or cytosine. For staining, cells were fixed with cold methanol for 10 min. After PBS washing, cells were treated with 2 N HCL for 30 min at 37 °C, followed by an incubation of 5 min with 0,1 M borate buffer at pH 8,5. After washing steps, the cells were incubated with medium B plus antibody or respective isotype controls for 1 h at room temperature.

Detection was performed by incubation with a FITC-conjugated, affinity purified F(ab0)2 fragment of goat anti-mouse or anti-rabbit IgG (Abcam, Cambridge, UK) for 1 h at room temperature in the dark. The labeled cells were analyzed by flow cytometry using a BD FACS Aria III (Becton & Dickinson, Mountain View, CA, USA). Histone modification and global DNA methylation associated to the cells was measured using FITC fluorochrome and calculated as mean fluorescence intensity (MFI) for each sample. The analyses are performed considering 100% untreated samples. 1 × 10^4^ events were acquired and histograms showed the mean ± SEM of the MFI values obtained from three independent experiments.

### Western blot assay

For Western blot analyses, cells were lysed in RIPA buffer (50 mM Tris-HCl pH 7.2, 150 mM NaCl, 1% NP40, 0.1% SDS, 0.5% DOC, 1 mM PMSF, 25 mM MgCl2, and supplemented with a phosphatase inhibitor cocktail). Protein concentration was determined by the BCA assay (Bio-Rad Laboratories, Hercules, CA). Equivalent amounts of protein (50 μg) were electrophoresed on 10% SDS–polyacrylamide gels. Precision Plus Protein™ Dual Color Standards (Bio-Rad) was used to determine molecular weight. Gel was electroblotted onto nitrocellulose membrane by using a Trans Blot Turbo system (Bio-Rad) following the manufacturer’s instructions. The membrane was blocked with 5% milk in TBS-0.1% Tween (TTBS) for 1 h at RT and washed with TTBS. Membrane was then incubated with specific primary anti-human antibodies against HDAC1 (1:5000; Abcam), HDAC2 (1:1000; Abcam), DNMT1 (1:250; Abcam), DNMT3a (1:500; Abcam) or GAPDH (1:5000; Millipore) over night at 4 °C. Total GAPDH antibody was used for assessing loading. Membrane was then washed with TTBS and incubated with the appropriate HRP-conjugated secondary antibody diluted 1:5000 in 3% milk in TTBS for 1 h at RT. Membrane was then washed three times with TTBS. Immunoreactive protein bands were visualized by the Pierce™ ECL Western Blotting Substrate (Thermo Scientific) according to the manufacturer’s instructions. The densitometric analyses were performed using Image J software and were expressed as ratio of GAPDH densitometry versus tested protein densitometry.

### Immunofluorescence

Treated and untreated cells were grown on coverslips and fixed in methanol. Cells were permeabilized with 1% TRITON X 100 in PBS and blocked using 1% BSA. Cells were stained with monoclonal anti-AcH3K9 (Abcam) and corresponding IgG negative controls. Cells were subsequently stained with FITC rabbit anti mouse secondary antibodies, counterstained with DAPI. Visualization was performed using an EVOS microscope. Fluorescence intensities from images of six randomly selected microscopic fields of cells were semi-quantitatively analyzed by densitometry using Image J software NIH Image.

### Wound-healing assay

The wound-healing assay was performed to measure two-dimensional movement. All cell fractions (untreated or treated) were cultured in 24-well plates at a density of 50.000 cells/well until confluence. A wound was created in the center of the cell monolayers by a sterile pipette tip. The phase contrast images were captured after 0, 4, 24 and 48 h. The analyses are performed considering 100% wound size at the time of culture and analyzed by Image J software. All experiments were performed in triplicates.

### Soft agar

To measure in vitro tumorigenicity due to drug treatments, treated and untreated cells at a density of 500 cells per well in 24-well plates were plated in soft agar, in triplicates. The test was performed using 0.8 and 0.3% agar in DMEM as the base and top layers, respectively. Cells were incubated for 21 days at 37 °C in a humidified atmosphere at 5% CO_2_ in air, and 50 ml of DMEM culture medium was added twice a week. At the end of the incubation period, colonies were stained with nitroblue tetrazolium (NBT, Sigma) at a concentration of 1 mg/2 ml in PBS and counted using an inverted microscope (Nikon TS 100, Milan, Italy). The colony efficiency was calculated as proportion of colonies per total number of seeded cells. The data were analyzed by Image Pro Plus software. All experiments were performed in triplicates.

### Real-time q-PCR

Total RNA was extracted using TRIzol Reagent (Invitrogen, Milan, Italy) according to the manufacturer’s protocol. RNA concentration and purity were determined by A260 and A260/A280 ratios, respectively. The integrity of total RNA was assessed on standard 1% agarose/formaldehyde gels. The RNA samples were treated with DNase I to remove residual traces of DNA. Expression levels of genes involved in angiogenesis were analyzed using real-time quantitative PCR. All real-time PCR reactions were performed using StepOne Thermocycler (Applied Biosystems, Monza, Italy) and the amplifications were done using the SYBR Green PCR Master Mix (Applied Biosystems, Monza, Italy). The thermal cycling conditions were: 50 °C for 2 min followed by an initial denaturation step at 95 °C for 2 min, 40 cycles at 95 °C for 30s, 60 °C for 30s and 72 °C for 30s. Real time q-PCR results were normalized to those of the housekeeping gene glyceraldehyde-3-phospate dehydrogenase (GAPDH). The primer sequences were the following (Additional file 1):

*Sox2*, 5-CGATGCCGACAAGAAAACTT-3 (sense) and 5-CAAACTTCCTGCAAAGCTCC-3 (antisense);

*Nanog*, 5-TTCAGTCTGGACACTGGCTG-3 (sense) and 5-CTCGCTGATTAGGCTCCAAC-3 (antisense);

*OCT3/4*, 5-ACATGTGTAAGCTGCGGCC-3 (sense) and 5-GTTGTGCATAGTCGCTGCTTG-3 (antisense);

*CD133*, 5- ATGACAAGCCCATCACAACA-3 (sense) and 5- CCTGAGTCACTACGTTGCCA-3 (antisense);

*SLUG*, 5-GAGCATTTGCAGACAGGTCA-3 (sense) and 5-CCTCATTGTTTGTGCAGGAGA-3 (antisense);

*OSTEOCALCIN*, 5- CTCACACTCCTCGCCCTATTG-3 (sense) and 5-CTTGGACACAAAGGCTGCAC-3 (antisense);

*TWIST*, 5-TCTCGGTCTGGAGGATGGAG-3 (sense) and 5-GTTATCCAGCTCCAGAGTCT-3 (antisense);

*VIMENTIN*, 5-CCTTGAACGCAAAGTGGAATC-3 (sense) and 5-GACATGCTGTTCCTGAATCTGAG-3 (antisense);

*E-CADHERIN*, 5-GGTCACAGCCACAGACGCGG-3 (sense) and 5- GGAAACTCTCTCGGTCCAGCCCA-3 (antisense);

*GAPDH*, 5-GGAGTCAACGGATTTGGTCG-3 (sense) and 5-CTTCCCGTTCTCAGCCTTGA-3 (antisense).

### Cell transfection

shHDAC2, and negative control shRNA (mock) neomycin-resistant Sure Silencing shRNA plasmids were purchased from SABioscience (Qiagen, Milano, Italy). The target sequence used against human *Hdac2* was as follows: 5’-TCAACCTAGTGCTGTGGTATT-3′. Negative control shRNA sequence was: 5’-GGAATCTATTCGATGCATAC-3′. MG63 and Saos2 cells were stably transfected with these constructs in an Amaxa Nucleofector device with the Amaxa Cell Line Nucleofector Kit V (Lonza GmbH, Cologne, Germany) and according to the manufacturer’s instructions. Clones with downregulated expression of HDAC2 were selected with 500 μg/ml G418. Clones were screened by flow cytometry and then analyzed for stemness markers expression by flow cytometry, sarcosphere-forming efficiency and in vitro tumorigenicity assay by soft agar.

### In vivo tumorigenicity by subcutaneous xenotransplantation into NOD/SCID IL2R-gamma mice

Mock MG63 and HDAC2 depleted-MG63 cells were injected subcutaneously into each flank of locally bred NOD/SCID IL2R-gamma-0 (NSG) mice [25, 26].

For this purpose, cells were enzymatically dissociated, diluted in PBS, mixed with Matrigel, and injected subcutaneously in mice. Mice were monitored every 5 days for the appearance of subcutaneous tumors. After 30 days, mice were sacrificed, and the tumor volume was calculated by the formula (l x w^2^)/2. The injection experiments were made in triplicate. All mouse experiments were performed according to the Institutional Animal Care and Use Committee procedures and guidelines of University of Campania.

#### Immunohistochemistry

Osteosarcoma paraffin-embedded tissue sections derived from 20 human biopsies were obtained from archival paraffin blocks. The sections were deparaffined and rehydrated with xylene, a decreasing scale of alcohols (100, 95, and 75°), and then distilled water. Immunohistochemical analyses for HDAC2 (Abcam) were performed with the Dako AEC kit, according to the manufacturer’s instructions. The nuclei were counterstained with hematoxylin, and the samples were observed under an inverted light microscope. The percentage of cells positive or negative for HDAC2 was scored as follows: negative = 0, positive staining < 10% = 1, positive staining ≥10 and < 33% = 2, positive staining ≥33 and < 66% = 3, positive staining ≥66% = 4. Intensity of staining was scored on a scale of 0–3: no color reaction = 0, mild reaction = 1, moderate reaction = 2, and intense reaction = 3. Immunoreactive score (IRS) was derived by multiplying immunoreactive cell scores and intensity of staining scores to compute an immunoreactive score ranging from 0 to 12.

### Statistical analysis

Values are shown as the mean ± S.E.M. of measurements of at least three independently performed experiments to avoid possible variation of cell cultures. Student’s t test was employed, and *p* < 0.05 was considered to be statistically significant.

## Results

### Investigating the effect of DAC and VPA on survival and growth of osteosarcoma cells using MTT assay

We investigate the possible epigenetic mechanism occurring in osteosarcoma by treating Saos2 and MG63 cells with VPA and DAC, alone or in combination of both. Therefore, we determined non-cytotoxic concentrations of both drugs using MTT and growth curve evaluation. MTT analyses performed 48 h post-treatment showed no significant differences in cell growth, while after 72 h (and using different concentrations of both drugs) there was high cytotoxicity. Furthermore, at 100 μM of DAC, cells were dead independently by treatment length. The use of VPA concentration of 0.5 mM up to 48 h did not induce differences in cell growth, whereas 1 mM VPA reduced cell growth in a time-dependent manner (Additional file 2). Cell growth curves analyses were also performed, using the above concentrations, without showing any statistically significant differences in cell proliferation at 48 h confirming data of MTT analyses (Additional file 2). Moreover, the calculation of IC50 showed that, both for Saos2 and MG63 cells, IC50 values were 10, 5 and 3 μM for 48,72 and 96 h, respectively for DAC treatment, and 0,7, 0,6 and 0,1 mM for 48,72 and 96 h respectively for VPA treatment. Being our aim that to evaluate the epigenetic alterations occurring in osteosarcoma, we selected the concentrations of 3 μM of DAC and 0,5 mM of VPA and their combination for 48 h for all further experiments.

### Treatments with VPA, DAC or their combination increased the expression of CD133, OCT4, SOX2 and NANOG in osteosarcoma cells

**Fig. 1:**
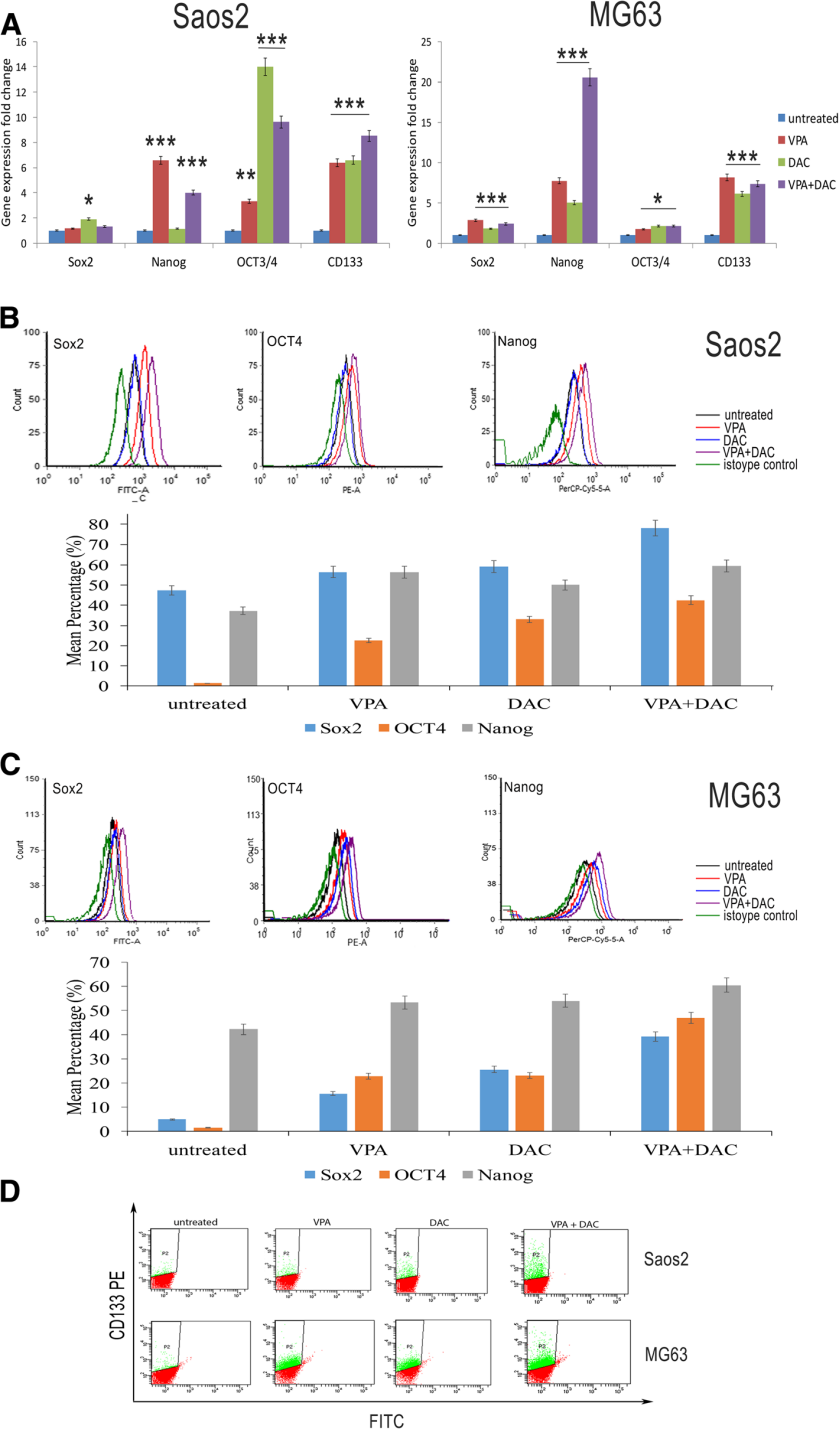
Evaluation of stemness factors on Saos2 and MG63 cell lines after VPA and DAC treatment. (**a**) real-time PCR for SOX2, NANOG, OCT4 and CD133 showing an increase of these genes after VPA and DAC treatments; (**b**) Flow cytometry analyses of increased expression of SOX2, OCT4 and NANOG in Saos2 and MG63 cells after VPA and DAC treatments; (**c**) up-regulation of CD133 on Saos2 and MG63 cells after VPA and DAC treatments analysed by flow cytometry. * *p* < 0.005, ** *p* < 0.001, *** *p* < 0.0001 compared to the untreated cells

To investigate the effect of DAC and VPA on osteosarcoma stem cells, we analyzed different markers of stemness by RT-PCR and flow cytometry. We performed a gene expression analysis for *OCT4*, *SOX2*, *NANOG* and *CD133*. Treatment of Saos2 cells with DAC induced an increase of *OCT4*, *SOX2* and *CD133*mRNA levels, but not of *NANOG* mRNA level. On the contrary, treatment with VPA induced an increase of *NANOG*, *OCT4* and *CD133* mRNA levels but not of *SOX2* mRNA levels. The combination of both drugs induced a strong increase of *OCT4* and *CD133* mRNA levels. VPA and DAC treatment on MG63 cells, another osteosarcoma cell line, induced an increase of all stemness genes when compared to those of untreated cells. Interestingly, drug combination led to a strong increase of *NANOG* mRNA levels (Fig. 1a). Flow cytometry analyses demonstrated that VPA and DAC induced an increase of SOX2, OCT4 and NANOG proteins, in both cell lines (Fig. 1b and Additional file 3). Remarkably, CD133 expression was increased after treatments both in Saos2 and MG63 cell lines. In particular, both VPA and DAC induced a considerable increase of CD133 expression and particularly in MG63 cells (Fig. 1c). The combination of the two drugs resulted in almost 3-fold increase of CD133 expression, when compared to untreated cells. Treatment with VPA or DAC induced a two-fold increase of CD133 expression and when compared to untreated cells (Additional file 4). In conclusion, DAC and VPA induced an increase of stemness as reflected by increased mRNA and protein levels of CD133, OCT4, Sox2 and NANOG.

### VPA, DAC treatments and their combination did not induce changes in the expression of EMT-related markers and in osteocalcin, CD29 and CD44 expression

VPA and DAC have been previously shown to induce epithelial to mesenchymal transition (EMT) [27, 28]. It has been shown that those transcription factors that induce EMT in epithelial cells are involved, for example, in drug resistance and other aggressiveness features of osteosarcoma [29]. Therefore, we determined the expression of SLUG, TWIST, vimentin, and e-cadherin, osteocalcin, CD29 and CD44 by real-time PCR and flow cytometry (Additional file 5).

VPA, DAC and their combination did not affect mRNA expression levels of vimentin, whereas VPA alone induced a strong increase of *TWIST* and e-cadherin mRNA levels in both cell lines when compared to untreated cells. DAC treatment led to a slightly increase of *TWIST* and a strong up-regulation of e-cadherin mRNA levels.

Treatment combining VPA and DAC induced only a strong increase of e-cadherin. Regarding to osteocalcin, treatments induced a decrease of mRNA levels compared to those of untreated cells (Additional file 5).

These results were partially confirmed by flow cytometry. Although it was possible to observe an increase of vimentin expression, this was not statistically significant in MG63 cells, whereas no change in its expression was detectable for Saos2 cells (Additional file 5) which confirms our observations on mRNA levels. Moreover, flow cytometry analyses showed that e-cadherin was undetectable in MG63 and Saos2 cells (Additional file 5). Osteocalcin, CD29 and CD44 expressions were not affected by treatments (almost 90% of expression). In summary and apart from inducing a slight increase of *TWIST*, DAC and VPA did not affect EMT. Similarly, no effect was observed for vimentin, osteocalcin, CD29 and CD44 expression following treatments.

### VPA, DAC alone and in combination increased sarcospheres formation rate and colonies formation in soft agar

**Fig. 2:**
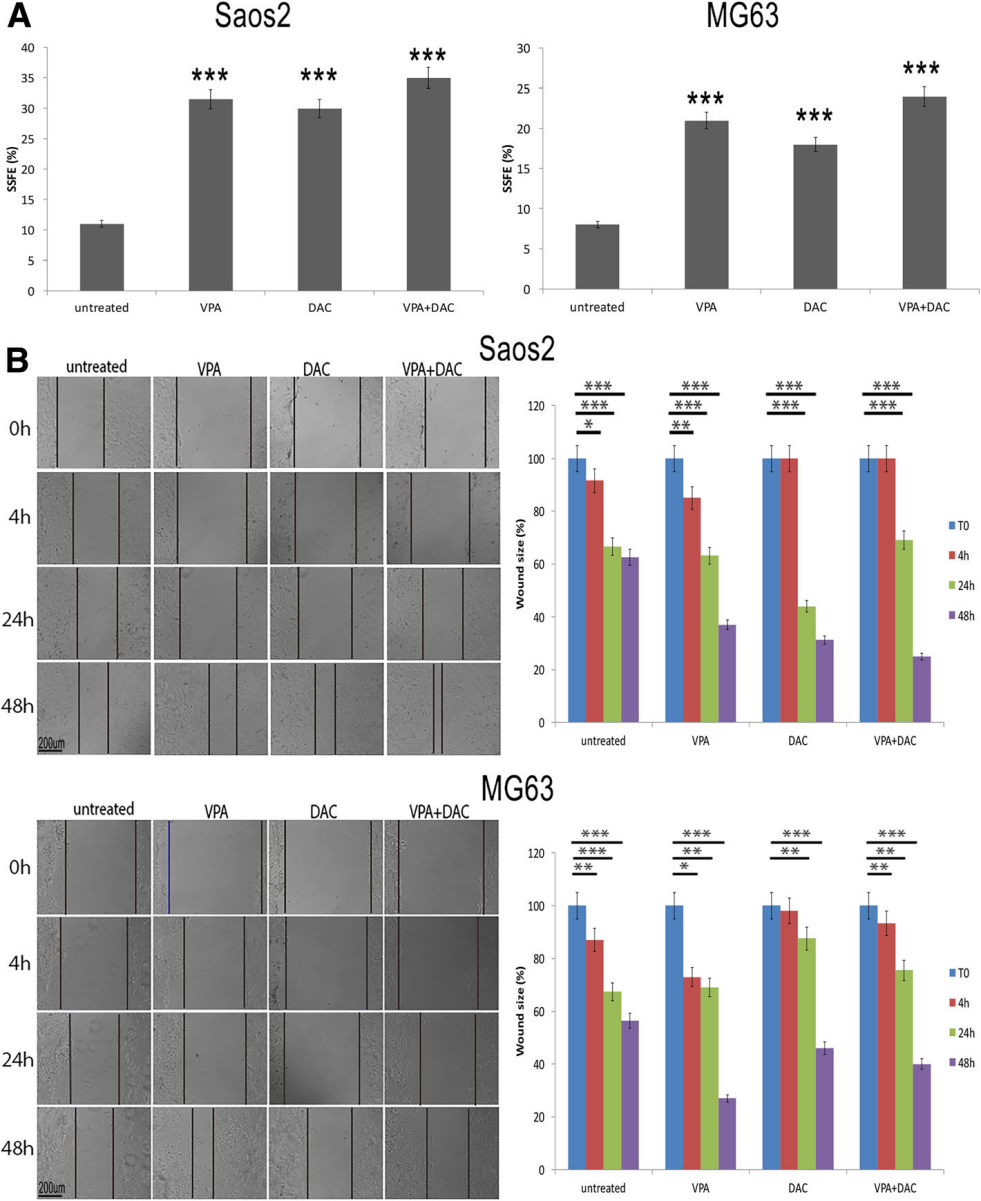
Analyses of efficiency of forming sarcospheres, and wound healing assay on Saos2 and MG63 cell lines after VPA and DAC treatment. (**a**) SSFE showing an increase of sarcospheres formation both in Saos2 and MG63cells after drug treatments; (**b**) Wound healing assay showing that VPA and DAC a decrease of wound size in treated cells. * *p* < 0.005, ** *p* < 0.001, *** *p* < 0.0001 compared to the untreated cells

Tumor stemness is also characterized by the ability to form spheres and colonies in soft agar. Therefore, we calculated the efficiency by which VPA and DAC induced sarcospheres and colonies formation. Both drugs led to an increase of sarcospheres formation rate, and when in combination, the rate was 3-fold increased (Fig. 2a). One method of analyzing the tumorigenic potential is the soft agar assay, which measures anchorage-independent growth. Assessment of growth kinetics revealed increased efficiency in colony-forming ability of treated cells and when compared to the untreated ones (Additional file 6). In particular, the combination induced a strong effect with a mean 3 fold-increase in colony-forming efficiency. Another characteristic that correlates with stemness is migration ability. Wound healing experiments to measure cell invasion revealed that both VPA and DAC enhanced motility of Saos2 and MG63 cells when compared to untreated controls and for 24 h. In this context, VPA induced a higher motility in MG63 compared to untreated and DAC treated cells. On the other hand, in Saos2 cells, drug combination increased cell migration, when compared to untreated and single agent VPA or DAC treated cells (Fig. 2b). Therefore, VPA, DAC and their combination enhanced the in vitro migration of investigated osteosarcoma cell lines.

### VPA, and DAC treatment affected histone modification and DNA methylation

**Fig. 3:**
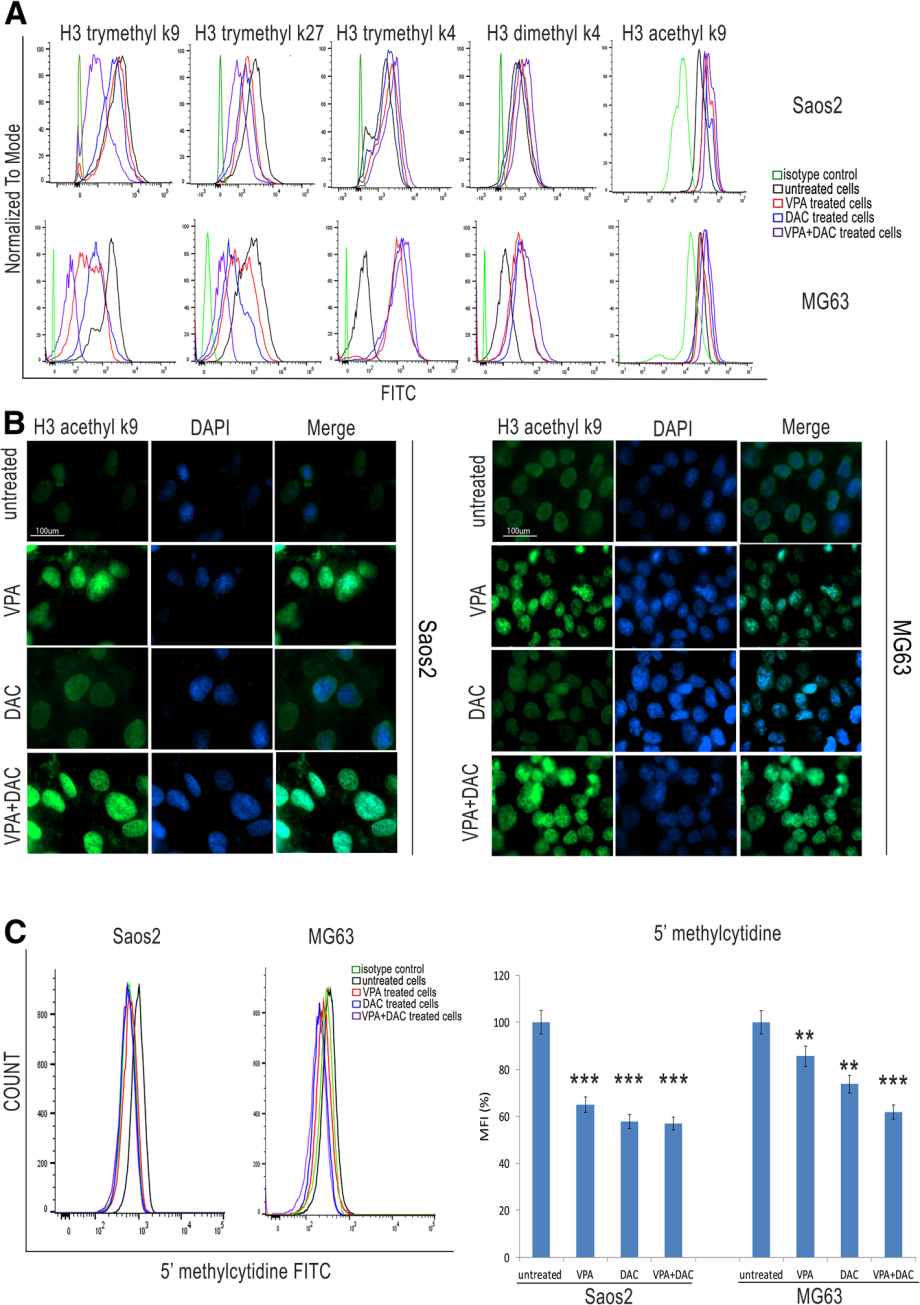
Evaluation of histone modifications, acetylation and global methylation of DNA on Saos2 and MG63 cell lines after VPA and DAC treatment. (**a**) Modulation of H3 trimethyl k9, H3 trimethyl k27, H3 trimethyl k4, H3 dimethyl k4 and H3 acetylation k9 by flow cytometry. VPA and DAC induced a decrease of H3 trimethyl k9 and H3 trimethyl k27, and an increase of H3 trimethyl k4, H3 dimethyl k4 and H3 acetylation k9; (**b**) Immunofluorescence for H3 acetylation k9 showing high levels of expression for VPA and DAC treatment; (**c**) Modulation of 5’methylcytidine showing a decrease of its expression after VPA and DAC treatment using flow cytometry. ** *p* < 0.001, *** *p* < 0.0001 compared to the untreated cells

**Fig. 4:**
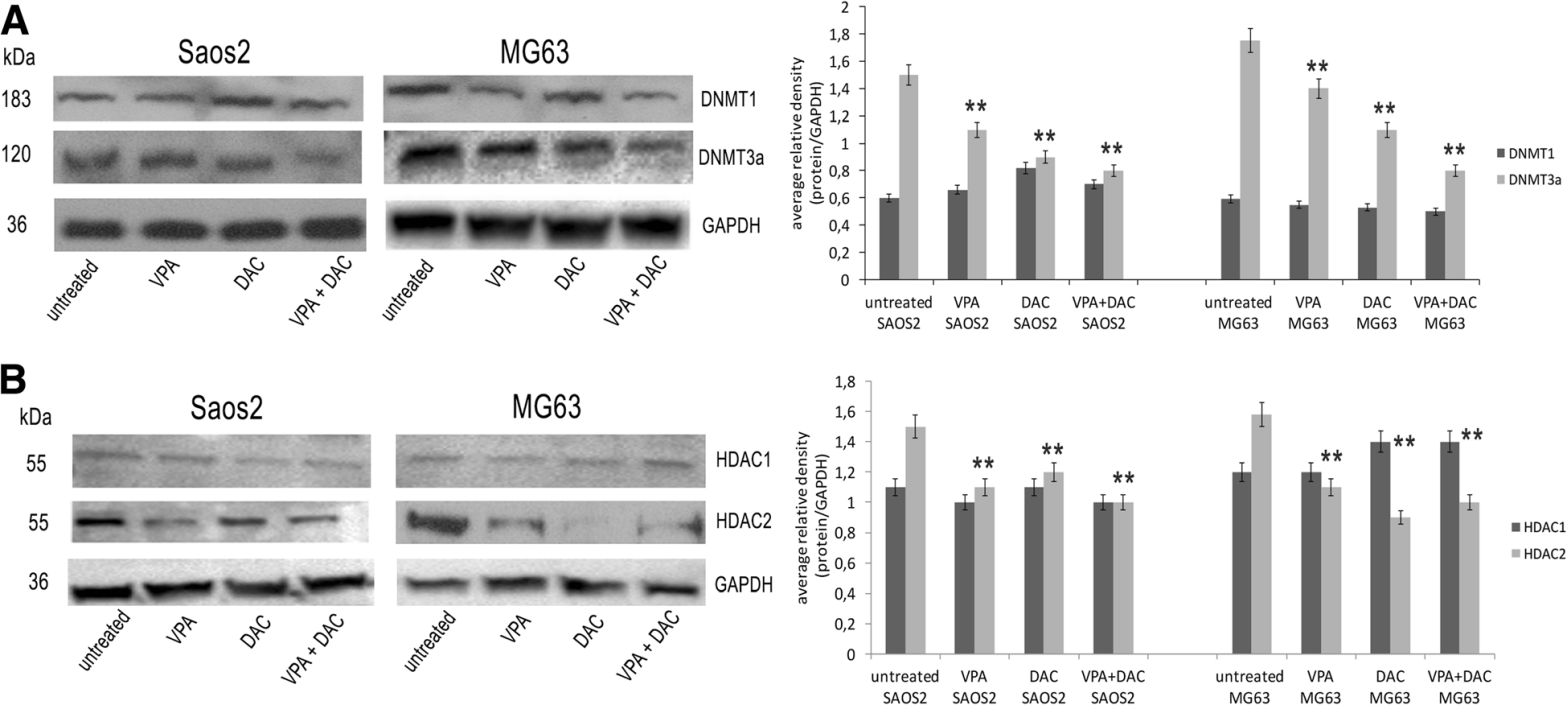
Analyses of DNMT1, DNMT3a, HDAC1 and HDAC2 on Saos2 and MG63 cell lines after VPA and DAC treatment. (**a**) Western blotting for DNMT1 and DNMT3a showing no change in the expression of DNMT1 and a decrease of DNMT3a expression; (**b**) Western blotting for HDAC1 and HDAC2 showing no change in the expression of HDAC1 and a decrease of HDAC2 expression. ** *p* < 0.001 compared to the untreated cells

To explore the landscape of histone modifications and DNA methylation that could be linked to our tumor stemness model and in response to VPA and DAC treatments, we performed an analysis of histone modifications that were previously shown to be involved in embryonic stemness, such as the repressive histone marks H3-trymethyl k9 (H3K9me3), H3-trymethyl k27 (H3K27me3), active histone marks H3-trymethyl k4 (H3K4me) and H3-dymethyl k4 (H3K4me2) [30]. Moreover, we investigated global epigenetic profiles of H3K9 acetylation and DNA methylation (Fig. 3). Flow cytometry analyses showed that VPA and DAC treatments, alone and in combination, induced a decrease of H3K9me3 and H3K27me3, and an increase of H3K4me2 and H3K4me3 (Fig. 3a). This indicates a correlation between those histone modifications and cancer stemness. These data were further confirmed by H3K9 acetylation and DNA methylation (Fig. 3). In fact, we found increased levels of H3K9 acetylation (*p* < 0.001) in treated cells, when compared with untreated cells (Fig. 3a). We also observed a substantial increase of acetylation for both cell lines when co-treated with VPA and DAC. These results were confirmed by immunofluorescence (Fig. 3b) semi-quantitatively analyzed by densitometry (additional file 7) where we observed increased H3K9 acetylation in treated cells as reflected by elevated fluorescence using the AcH3K9 antibody and when compared to untreated counterparts. Moreover, the levels of 5′ Methylcytidine, analyzed by flow cytometry, showed a significant reduction of global DNA methylation (*p* < 0.001) in treated cells and when compared with untreated cells (Fig. 3c). To better investigate this event, we performed a western blotting for DNA methyltransferases 1 and 3a (DNMT1, DNMT3a) that are the main enzymes involved in global DNA methylation [31] (Fig. 4a). In Saos2 and MG63 cell lines, DNMT1 expression did not change after drug treatment, whereas DNMT3a expression severely decreased (Fig. 4a). This is a very interesting finding, as it highlights that DNMT3a could be a key factor involved in cancer stemness in osteosarcoma carcinogenesis. VPA has been suggested to inhibit the activity of histone deacetylases 1 and 2 (HDAC1, 2) [32]. Therefore, we investigated the expression of the two enzymes after treatment in order to investigate their possible role in cancer stem cells. Western blotting analyses showed that in Saos2 and MG63 cell lines HDAC1 expression did not change after treatment, therefore HDAC1 does not appear to be involved in acetylation processes. However, HDAC2 levels decreased in both cell lines after treatment, mainly following VPA treatment and drug combination (Fig. 4b).

### HDAC2 knockdown promoted cancer stemness in osteosarcoma and tumor growth in mouse xenografts

**Fig. 5:**
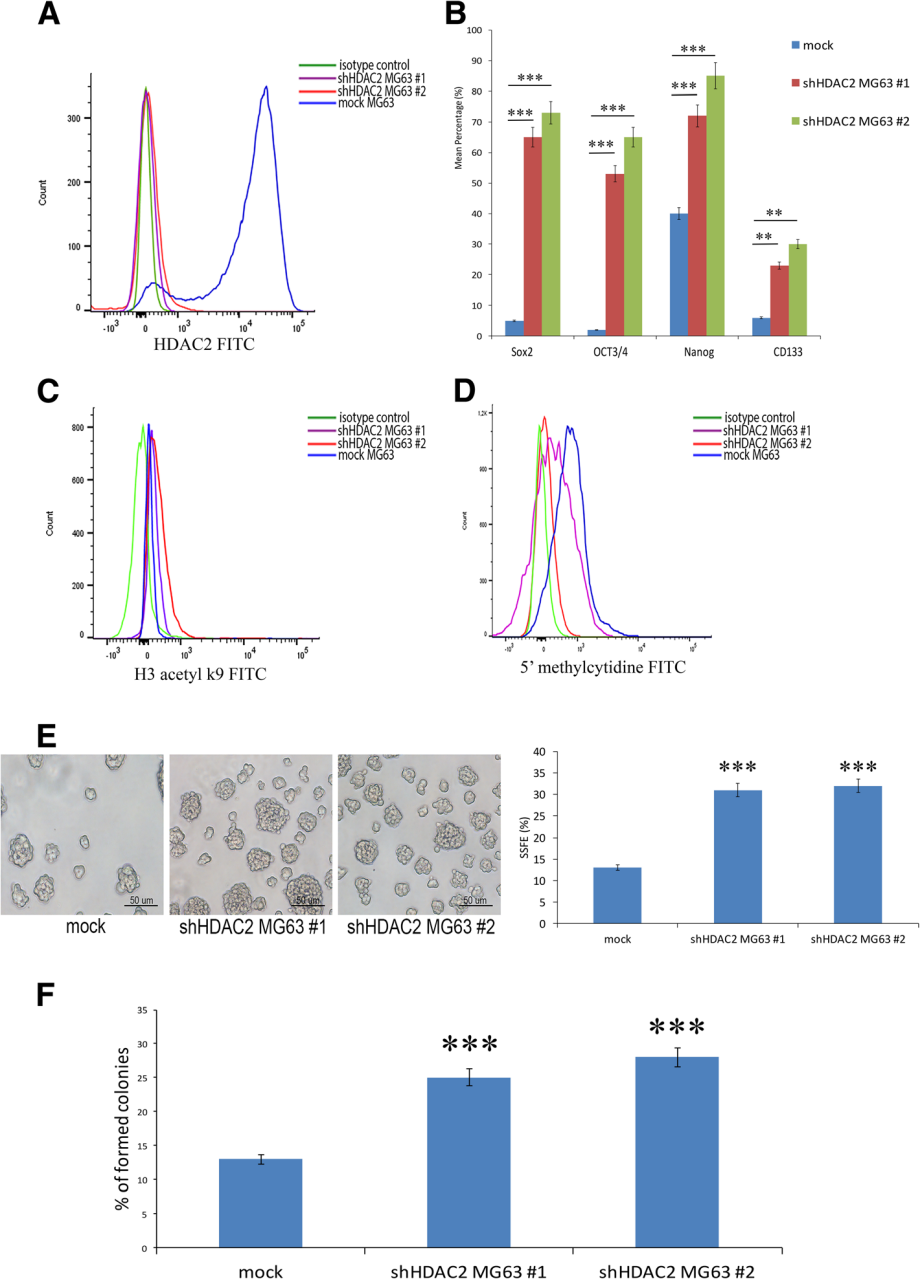
Analyses of stemness on shHDAC2 MG63 cell line. (**a**) Flow cytometry analysis of HDAC2 expression in shHDAC2 MG63#1 and 2 cells and MG63 mock after silencing; (**b**) Cytometric analysis of the expression of stemness factors in shHDAC2 MG63#1 and 2 cells showing a strong increase of SOX2, OCT4, NANOG and CD133 stemness markers; (**c**) Flow cytometry showing elevated expression of H3 acetyl k9 on shHDAC2 MG63#1 and 2 cells compared to mock cells; (**d**) Decrease of DNA methylation of shHDAC2 MG63#1 and 2 cells; (**e**) SSFE showing an increase of sarcospheres formation in shHDAC2 MG63#1 and 2 cells; (**f**) Soft agar assay showing increased ability to form colonies of shHDAC2 MG63#1 and 2 cells compared to those of mock cells. ** *p* < 0.001, *** *p* < 0.0001 compared to mock cells

To confirm our results regarding the involvement of HDAC2 in promoting cancer stemness, we performed a silencing of HDAC2 using HDAC2 shRNA both in MG63 (Fig. 5a) and Saos2 cells (Additional file 8). HDAC2 depleted-MG63 and Saos2 cells showed an increase of stemness markers CD133, OCT4, SOX2 and NANOG when compared to control cells (Fig. 5b and Additional file 8). This result was comparable to that observed following VPA and DAC treatments. Moreover, we found that HDAC2 depleted-MG63 and Saos2 cells had an increased H3K9 acetylation (Fig. 5c and Additional file 8) and a decreased global DNA methylation (Fig. 5d and Additional file 8). This observation also confirms the results obtained following VPA and DAC treatments. Interestingly, an increase in sarcospheres (Fig. 5e and Additional file 8) and colonies formation rates (Fig. 5f and Additional file 8) was also detectable for HDAC2 depleted-MG63 and Saos2 cells and compared to mock cells, which further supports the idea that HDAC2 Knockdown was effective in promoting stemness of osteosarcoma cells (Fig. 5 and Additional file 8).

**Fig. 6:**
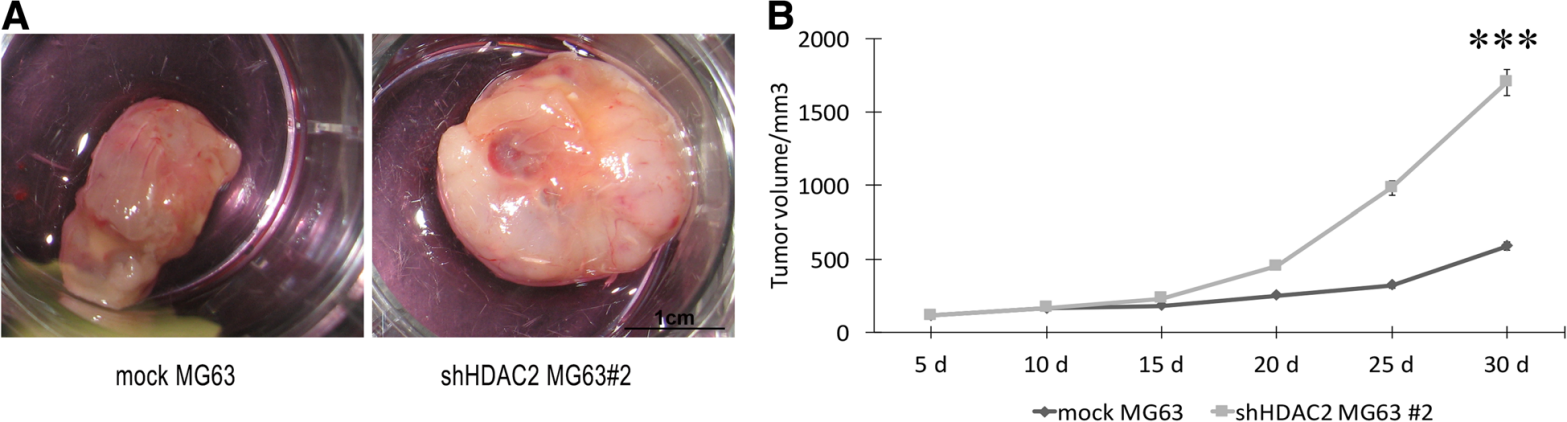
HDAC2 knowdown supported osteosarcoma tumorigenesis. (**a**) MG63 cells transfected with HDAC2 showed a tumor size grater than those of mock MG63 cells; (**b**) evaluation of tumor volume derived from an injection of sh HDAC2 MG63 cells compared to those of mock MG63 cells Data are presented as mean ± s.d. *** *p* < 0.0001 compared to mock cells

In addition, to investigate the in vivo effects of HDAC2 downregulation on osteosarcoma tumorigenesis, we injected knocked down HDAC2-MG63 cells in mice. We found that the HDAC2 downregulation significantly promoted tumor growth in nude mice compared to mock MG63 cells (Fig.6a) with a tumor size of 2,9 ± 0,5 fold greater than those detected in mock MG63 cells (Fig. 6b). These results indicate that HDAC2 Knockdown may promote the osteosarcoma tumorigenesis.

#### HDAC2 expression in human osteosarcoma biopsies

**Fig. 7:**
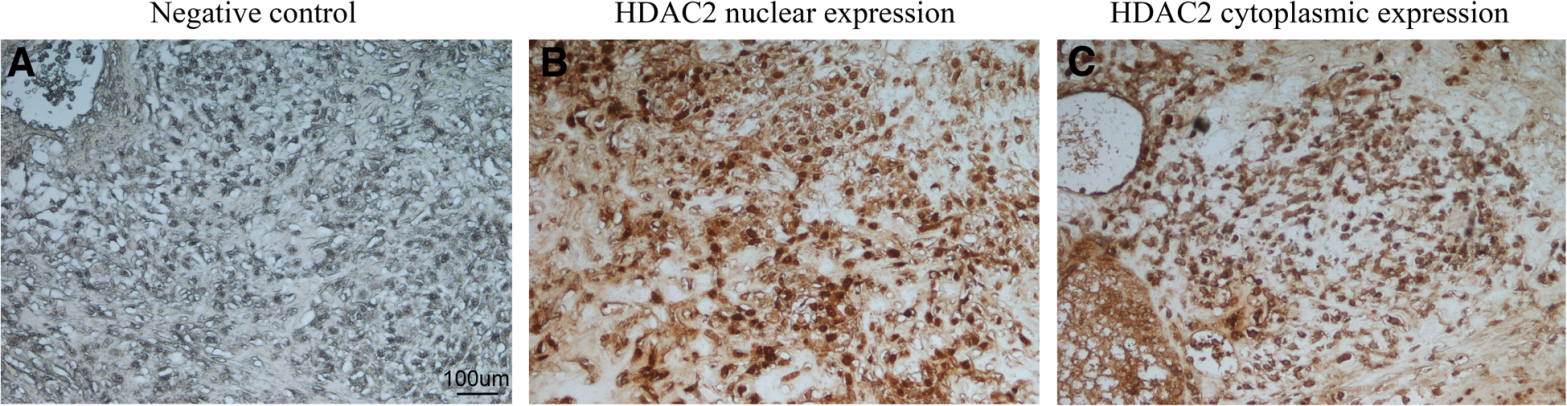
Immuohistochemical staining for HDAC2 in human osteosarcoma tissues. (**a**) negative control; (**b**) nuclear staining of HDAC2 with immunoreactive score of 12; (**c**); cytoplasmic staining of HDAC2 with immunoreactive score of 10

Immunohistochemical analyses of paraffin-embedded tumor samples confirmed the presence of HDAC2 in 95% of cells and in all cases tested (Fig.7 a,b). In particular, HDAC2 was mainly expressed in nuclei of osteosarcoma cells with an immunoreactive score > 7 (Fig.7b). Although this, cytoplasmic localization was also found and HDAC2 distribution was predominantly perinuclear (Fig.7c). Also in this case, immunoreactive score was > 7.

## Discussion

Osteosarcoma is a disease of differentiation characterized by a variety of molecular and cytogenetic alterations, including severe chromosomal rearrangements [30–33]. The loss of differentiation is correlated with high grade osteosarcoma, whereas a more differentiated phenotype is linked to low grade osteosarcoma [34]. The mechanisms that are involved in the correlation between aberrant differentiation and carcinogenesis and in the synergy between genetic and epigenetic processes might be decisive in maintaining osteosarcoma cells in undifferentiated state. Several studies have shown that DNA demethylation and the inhibition of HDACs induce CSCs maintenance and may lead to reprogramming differentiated cells into stem cells [19, 33, 34]. Here, we investigated the epigenetic alterations occurring in osteosarcoma, treating Saos2 and MG63 cells with DAC and VPA and identified a possible molecular mechanism in stemness maintenance. For this purpose, we examined stemness and EMT related-markers expression, sarcospheres and colonies formation efficiency, invasion abilities, global methylation status and specific histone modifications. We demonstrated that treating osteosarcoma cell lines with VPA and DAC promotes the expression of the stem cell factors OCT4, NANOG, SOX2 and CD133 [35, 36] and induces an increase of the spheres and colonies formation efficiency, reinforcing the idea that the said two substances are capable to maintain the stemness phenotype.

Recent studies reported that VPA and DAC can also induce EMT [27, 28]. In cancer, EMT enables cancer cells to migrate and form metastasis by switching the phenotype from epithelial to mesenchymal profile [28, 37]. EMT, as well as, cancer stemness phenotype, is also characterized by an increase of migratory and invasive abilities. In osteosarcomas, it has been demonstrated that TWIST1 expression provides osteosarcoma cells resistance to chemotherapy, whereas downregulation of SLUG induces a decrease of migration [37, 38]. In our study, following drug treatments, we observed that expression of vimentin, CD44, CD29 and osteocalcin remained similar, the e-cadherin levels were negative and the cell shape did not change. Only Twist and SLUG mRNA levels increased. Moreover, both drugs induced an increase of invasion ability. All the above is in line with the so called “collective cell migration phenomena” where cells do not undergo to distinct and mutually exclusive morphological categories, but rather they are a general continuum of morphological variety which can be obtained combining diverse and complementary mechanisms as previously proposed by Campbell et al. in 2016 [39].

Having defined the stemness characteristics of osteosarcoma cells in response to VPA and DAC treatments, our interest was devoted to determine the epigenetic events characterizing osteosarcoma CSCs. Therefore, we investigated specific histone modifications and global DNA methylation. It is widely known that the acetylation of histones H3 and H4 is mainly associated with gene expression and the methylation of H3 lysine 4, which recruits chromatin-remodeling enzymes and leads to a relaxed or “open” chromatin structure that is permissive to transcription [40]. On the contrary, DNA methylation, trimethylation of H3 lysine 9 and lysine 27 elicits the compaction of chromatin leading to gene silencing [38]. Moreover, it has been demonstrated that embryonic stem cells possess the so-called bivalent domains of chromatin that contains coextisting active (H3K4me3) and repressive (H3K27me3) marks at promoters of specific genes involved in the development [41, 42]. Notably, we showed that repressive histone marks decreased and active histone marks increased after drugs treatment. These histone modifications were also associated with an increase of acetylation of histones H3 and a decrease of DNA global methylation. Therefore, here we demonstrate, for the first time, that the stemness profile of osteosarcoma cells is characterized by DNA hypomethylation, increase of H3 histone acetylation, H3K4me2 and H3K4me3 and decrease of H3K9me3 and H3K27me3. Histone modification and methylation status of DNA are dynamically orchestrated by enzymes that remove or add covalent modifications to histone proteins and CpG islands [30].

HDACs are enzymes that reduce the level of acetylation of core histones, repressing gene transcription [30] and DNA methylation patterns are generated and maintained by the activity of specific enzymes: DNMTs [30].

Here, we investigate the effect of VPA and DAC on HDAC1 and HDAC2 that usually are inhibited by VPA and DNMT1 and DNMT3a that are targeted by DAC. We observed that HDAC1 and DNMT1 expressions remained similar independently by treatments indicating that they have no key role in the processes of acetylation and methylation in osteosarcoma. Conversely, we found that VPA and DAC affected HDAC2 and DNMT3a expressions. Therefore, to reinforce our hypothesis, we performed a HDAC2 silencing in MG63 and Saos2 cells and investigated their stemness profile and their ability to form new tumors in mice. Unfortunately, a silencing also for DNMT3a cannot be performed because it highly prevents osteosarcoma cells viability. MG63 and Saos2 shHDAC2 cells showed a stemness profile resembling to the effect observed with drug treatments. In fact, the lack of HDAC2 promoted an increase of Sox2, OCT4, Nanog and CD133, a decrease of DNA methylation, an increase of acetylation and colonies formation. Moreover, MG63 shHDAC2 cells were able to generate tumors that grew faster and were greater than those originating from mock MG63. In parallel, we investigated HDAC2 expression in human osteosarcoma tissues and we found that HDAC2 was expressed in all cases and it was mainly localized in nucleus. Moreover, some osteosarcomas cells showed also a cytoplasmic distribution of HDAC2 reinforcing our idea that HDAC2 alteration could be an important event in tumorigenesis of osteosarcoma.

Then, we identify for the first time two enzymes, HDAC2 and DNMT3a, that have a key role in maintaining stemness status of osteosarcoma cells sustaining also the tumor growth in vivo*.*

**Fig. 8:**
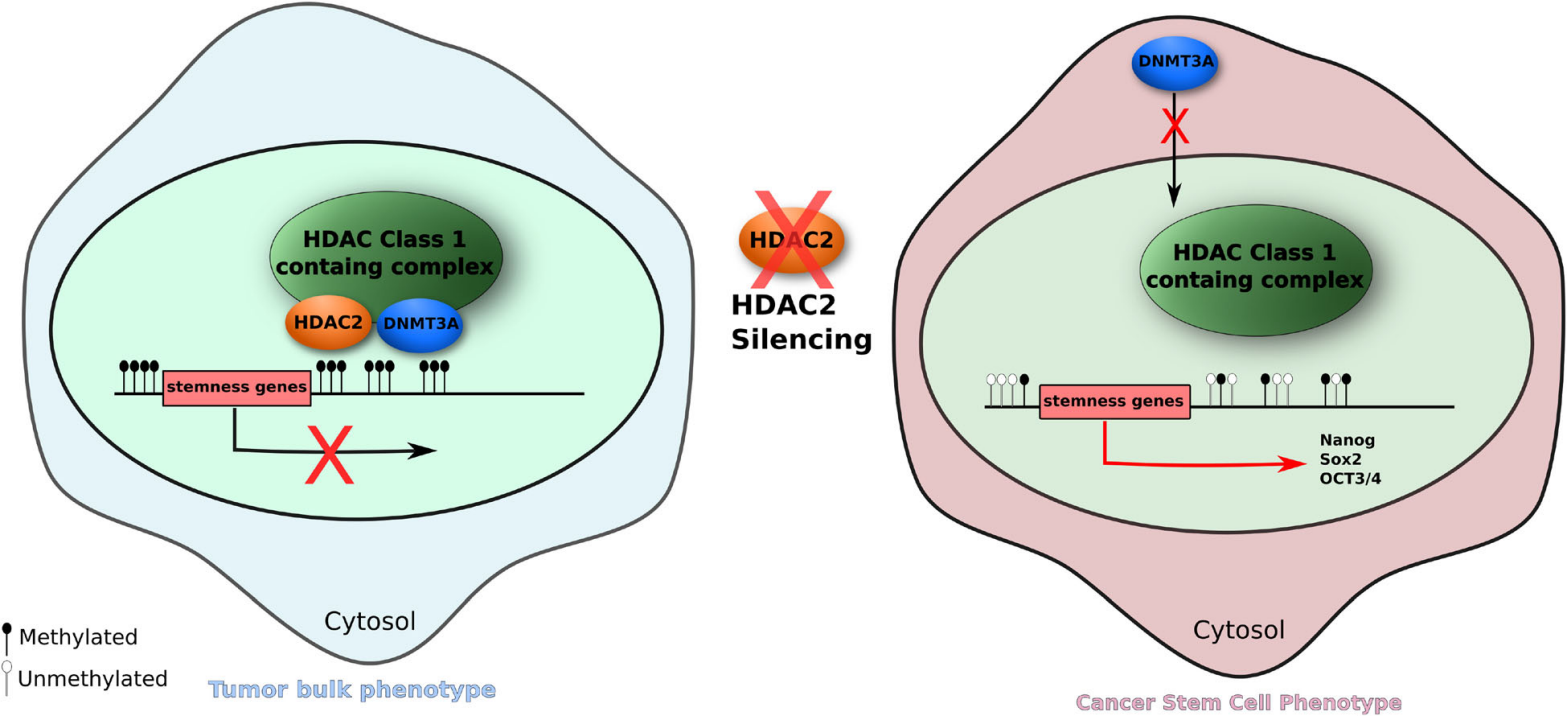
Proposed mechanistic model of HDAC2 function in osteosarcoma. Decrease of HDAC2 or DNMT3a expression or loss of both inhibits the formation of HDAC2/DNMT3a complex leading to transcription of genes involved in osteosarcomas stemness and promoting tumor initiation and progression

It is well known that histone modifications and DNA methylation interact at multiple levels to regulate gene expression status. In particular, DNMTs are able to recruit HDACs achieving gene silencing and chromatin condensation. For instance, it has been demonstrated that DNA methylation can induce H3K9 methylation by specific proteins, such as MeCP2, leading to a gene repression [43–45]. In our study, we can hypothesize that in normal conditions, HDAC2 can recruit DNMT3a from cytosol to the nucleus. This complex can induce gene repression, leading to a differentiated phenotype. Conversely, a decrease of HDAC2 or DNMT3a expression or loss of both inhibits the formation of HDAC2/DNMT3a complex leading to transcription of genes involved in osteosarcoma stemness (Fig. 8). These preliminary results suggest that targeting of HDAC2 could have a strong potential therapeutic implication in the treatment of human osteosarcoma.

## Conclusions

In summary, our preliminary study highlights peculiar features of osteosarcoma CSCs biology as follows: i. VPA and DAC induce an expansion of CSCs; ii. epigenetic pattern of such cells is characterized by DNA hypomethylation, increase of H3 histone acetylation, H3K4me2 and H3K4me3 and decrease of H3K9me3 and H3K27me3; iii. HDAC2 and DNMT3a are key enzymes in generating cancer stem phenotype in osteosarcoma; iv. induction of HDAC2 expression could have a strong potential therapeutic implication. Therefore, our study identifies, for the first time, specific epigenetic events that maintain cells in undifferentiated state. This is crucial in osteosarcoma treatment since the identification of specific epigenetic defects can support the design of better therapeutic strategies leading to the development of target epigenetic drugs. Therefore, the combination of epigenetic therapeutic approaches with standard chemotherapy could be a promising strategy for successful treatment of osteosarcoma. Moreover, these approaches show the advantage to sensitize cancer cells, in particular cancer stem cells, that are refractory to standard chemotherapy.

## Additional files

Additional file 1: **Table S1.** Primer sequences. (DOC 34 kb)

Additional file 2: **Figure S1.** Evaluation of on Saos2 and MG63 cells viability after VPA and DAC treatment. (a) MTT assay showing effect of VPA and DAC on Saos2 and MG63 cells; (b) Growth curves of osteosarcoma cells after drug treatments. (TIF 16532 kb)

Additional file 3: **Table S2.** Distribution of Sox2, OCT4 and Nanog stemness markers in Saos2 and MG63 cells after drug treatments. (DOC 32 kb)

Additional file 4: **Figure S2.** Evaluation of CD133 marker on Saos2 and MG63 cell lines after VPA and DAC treatment. Flow cytometric analyses showed a strong increase of CD133 expression after drug treatments. ** *p* < 0.001, *** *p* < 0.0001 compared to the untreated cells. (TIF 242 kb)

Additional file 5: **Figure S3.** Analyses of EMT related markers on Saos2 and MG63 cell lines after VPA and DAC treatment. (a) Real-time PCR for SLUG, TWIST, Vimentin E-cadherin and Osteocalcin showing an increase of SLUG mRNA levels in MG63 and Saos2 cells following VPA and combination treatment, a strong increase of TWIST mRNA levels in both cell lines following VPA treatment and an increase of E-cadherin mRNA after DAC treatments. Vimentin mRNA levels did not change. Osteocalcin mRNA levels decreased in both cell lines; (b) Expression of CD324, vimentin and osteocalcin in Saos2 and MG63 cell lines after VPA and DAC treatment analysed by flow cytometry. CD324, Vimentin and Osteocalcin did not change after drug treatment compared to untreated cells. * *p* < 0.005, ** *p* < 0.001, *** *p* < 0.0001 compared to the untreated cells. (TIF 8966 kb)

Additional file 6: **Table S3.** Primary colony-forming efficiency of treated versus untreated cells in Saos2 and MG63 cell lines. (DOC 36 kb)

Additional file 7: Fluorescence evaluation. Densitometry analyzing semi-quantitatively fluorescence for H3 acetylation k9. Data showed high levels of expression following VPA and DAC treatments. (TIF 147 kb)

Additional file 8: Analyses of stemness on shHDAC2 Saos2 cell line. (**a**) Cytometric analysis of HDAC2 expression in shHDAC2 Saos2#1 cells and mock after silencing; (**b**) strong increase of SOX2, OCT4, NANOG and CD133 stemness markers expression in shHDAC2 Saos2#1 cells by flow cytometry; (**c**) elevated expression of H3 acetyl k9 on shHDAC2 Saos2#1 cells by flow cytometry; (**d**) decrease of DNA methylation of shHDAC2 Saos2#1 cells; (**e**) SSFE showing an increase of sarcospheres formation in shHDAC2 Saos2#1 cells; (**f**) Soft agar assay showing increased ability to form colonies of shHDAC2 Saos2#1 cells compared to those of mock cells. ** *p* < 0.001, *** *p* < 0.0001 compared to mock cells. (TIF 17257 kb)

## Abbreviations

bFGF: Basal fibroblast growth factor
CSCs: Cancer stem cells
DAC: 5’azacytidine
DNMT: DNA methyltransferase
EGF: Epidermal growth factor
HDAC: Histone deacetylase
MTT: 3-(4, 5-dimethylthiazol-2-yl)-2, 5-diphenyltetrazolium bromide
VPA: Valproic acid
