# Supplementary material for: HDAC2 depletion promotes osteosarcoma’s stemness both in vitro and in vivo: a study on a putative new target for CSCs directed therapy
Source: J Exp Clin Cancer Res. 2018 Dec 3;37:296. doi: 10.1186/s13046-018-0978-x (PMC6276256; doi:10.1186/s13046-018-0978-x)
Supplement: Supplementary file 1 — Table S1. Primer sequences. (DOC 34 kb) [file 13046_2018_978_MOESM1_ESM.doc]

| **Supplementary Table 1.** Primer Sequences | | |
| --- | --- | --- |
| Gene | Primer sequence | Ta/°C |
| SOX-2 | Fw: CGATGCCGACAAGAAAACTT  Rev: CAAACTTCCTGCAAAGCTCC | 58 |
| NANOG | Fw: TTCAGTCTGGACACTGGCTG  Rev: CTCGCTGATTAGGCTCCAAC | 58 |
| OCT3/4 | Fw: ACATGTGTAAGCTGCGGCC  Rev: GTTGTGCATAGTCGCTGCTTG | 58 |
| CD133 | Fw: ATGACAAGCCCATCACAACA Rev: CCTGAGTCACTACGTTGCCA | 58 |
| SLUG | Fw: GAGCATTTGCAGACAGGTCA  Rev: CCTCATTGTTTGTGCAGGAGA | 58 |
| OSTEOCALCIN | Fw: ctcacactcctcgccctattg Rev: cttggacacaaaggctgcac | 57 |
| TWIST | Fw: TCTCGGTCTGGAGGATGGAG  Rev: GTTATCCAGCTCCAGAGTCT | 58 |
| VIMENTIN | Fw: CCTTGAACGCAAAGTGGAATC Rev: GACATGCTGTTCCTGAATCTGAG | 58 |
| E-CADHERIN | Fw: GGTCACAGCCACAGACGCGG  Rev: GGAAACTCTCTCGGTCCAGCCCA | 60 |
| GAPDH | Fw: GGAGTCAACGGATTTGGTCG  Rev: CTTCCCGTTCTCAGCCTTGA | 57 |
