# Supplementary material for: HDAC2 depletion promotes osteosarcoma’s stemness both in vitro and in vivo: a study on a putative new target for CSCs directed therapy
Source: J Exp Clin Cancer Res. 2018 Dec 3;37:296. doi: 10.1186/s13046-018-0978-x (PMC6276256; doi:10.1186/s13046-018-0978-x)
Supplement: Supplementary file 3 — Table S2. Distribution of Sox2, OCT4 and Nanog stemness markers in Saos2 and MG63 cells after drug treatments. (DOC 32 kb) [file 13046_2018_978_MOESM3_ESM.doc]

| **Supplementary Table 2**. Distribution of Sox2, OCT4 and Nanog stemness markers in Saos2 and MG63 cells after drug treatments. | | | | |
| --- | --- | --- | --- | --- |
|  |  |  |  |  |
|  |  | Sox2 expression | OCT4 expression | Nanog expression |
|  |  | mean percentage (%) | | |
| Saos2 | untreated | 47,5±1,2 | 1,4±0,1 | 37,29±1,3 |
|  | VPA | 56,5±1,1 | 22,67±1,1 | 56,41±1,4 |
|  | DAC | 59,1±1,3 | 33,01±1,6 | 50,12±1,1 |
|  | VPA+DAC | 78±1,5 | 42,59±1,4 | 59,6±2,1 |
| MG63 | untreated | 4,97±0,5 | 1,65±0,7 | 42,3±1,5 |
|  | VPA | 15,66±0,7 | 22,9±1,1 | 53,41±1,7 |
|  | DAC | 25,71±1,3 | 23,2±1,5 | 54,1±1,7 |
|  | VPA+DAC | 39,33±2,1 | 46,89±1,8 | 60,6±2,1 |
