# Supplementary figures and images for: HDAC2 depletion promotes osteosarcoma’s stemness both in vitro and in vivo: a study on a putative new target for CSCs directed therapy
Source: J Exp Clin Cancer Res. 2018 Dec 3;37:296. doi: 10.1186/s13046-018-0978-x (PMC6276256; doi:10.1186/s13046-018-0978-x)

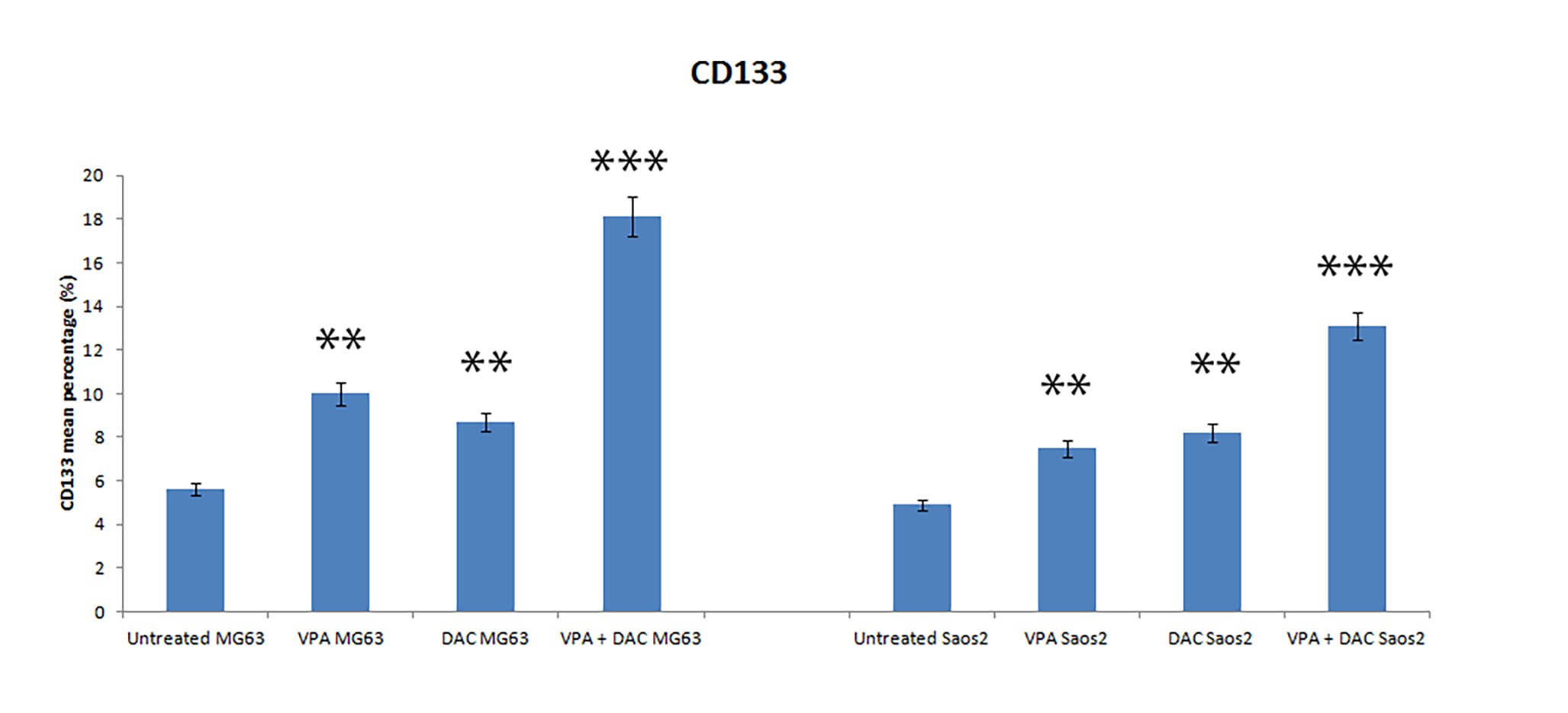

Supplement: Supplementary file 4 — Figure S2. Evaluation of CD133 marker on Saos2 and MG63 cell lines after VPA and DAC treatment. Flow cytometric analyses showed a strong increase of CD133 expression after drug treatments. ** p < 0.001, *** p < 0.0001 compared to the untreated cells. (TIF 242 kb) [file 13046_2018_978_MOESM4_ESM.tif]

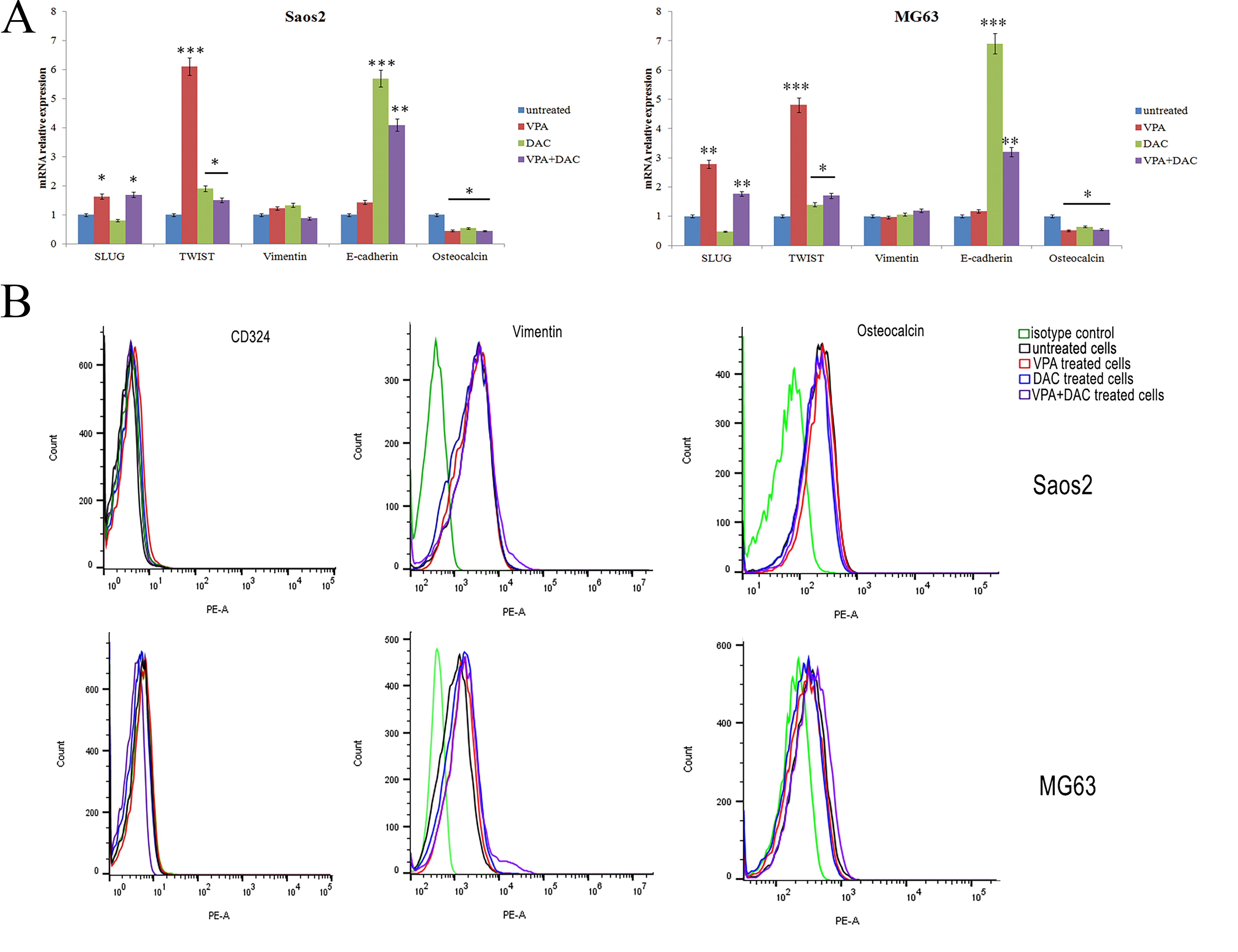

Supplement: Supplementary file 5 — Figure S3. Analyses of EMT related markers on Saos2 and MG63 cell lines after VPA and DAC treatment. (a) Real-time PCR for SLUG, TWIST, Vimentin E-cadherin and Osteocalcin showing an increase of SLUG mRNA levels in MG63 and Saos2 cells following VPA and combination treatment, a strong increase of TWIST mRNA levels in both cell lines following VPA treatment and an increase of E-cadherin mRNA after DAC treatments. Vimentin mRNA levels did not change. Osteocalcin mRNA levels decreased in both cell lines; (b) Expression of CD324, vimentin and osteocalcin in Saos2 and MG63 cell lines after VPA and DAC treatment analysed by flow cytometry. CD324, Vimentin and Osteocalcin did not change after drug treatment compared to untreated cells. * p < 0.005, ** p < 0.001, *** p < 0.0001 compared to the untreated cells. (TIF 8966 kb) [file 13046_2018_978_MOESM5_ESM.tif]

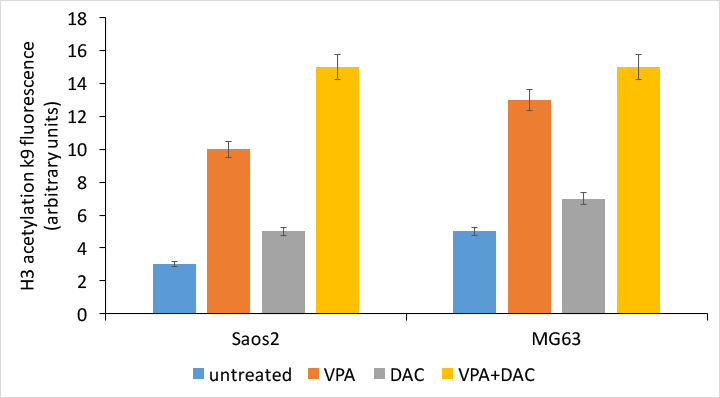

Supplement: Supplementary file 7 — Fluorescence evaluation. Densitometry analyzing semi-quantitatively fluorescence for H3 acetylation k9. Data showed high levels of expression following VPA and DAC treatments. (TIF 147 kb) [file 13046_2018_978_MOESM7_ESM.tif]
