# Supplementary material for: HDAC2 depletion promotes osteosarcoma’s stemness both in vitro and in vivo: a study on a putative new target for CSCs directed therapy
Source: J Exp Clin Cancer Res. 2018 Dec 3;37:296. doi: 10.1186/s13046-018-0978-x (PMC6276256; doi:10.1186/s13046-018-0978-x)
Supplement: Supplementary file 6 — Table S3. Primary colony-forming efficiency of treated versus untreated cells in Saos2 and MG63 cell lines. (DOC 36 kb) [file 13046_2018_978_MOESM6_ESM.doc]

| **Supplementary Table 3**. Primary colony-forming efficiency of treated versus untreated cells in Saos2 and MG63 cell lines. | | | | | |
| --- | --- | --- | --- | --- | --- |
|  |  |  |  |  |  |
|  |  | N° colonies | % of formed colonies | Fold increase | p-value |
| Saos2 | untreated | 5,7 ± 1,8 | 11,4±0,8 |  |  |
|  | VPA | 8,5±0,9 | 17±1,2 | 1,49 | 0,05 |
|  | DAC | 7,9±1,2 | 15,8±1,6 | 1,38 | 0,01 |
|  | VPA+DAC | 16,4±1,3 | 32±1,7 | 2,87 | 0,001 |
| MG63 | untreated | 7,5 ± 1,5 | 15±1,1 |  |  |
|  | VPA | 11,6±0,5 | 23,2±1,3 | 1,54 | 0,002 |
|  | DAC | 12,7±1,1 | 25,4±1,1 | 1,69 | 0,002 |
|  | VPA+DAC | 22,8±1,6 | 45,6±1,9 | 3,04 | 0,0001 |
